# Carbon-Starvation Induces Cross-Resistance to Thermal, Acid, and Oxidative Stress in *Serratia marcescens*

**DOI:** 10.3390/microorganisms3040746

**Published:** 2015-10-26

**Authors:** Joseph R. Pittman, La’Kesha C. Kline, William J. Kenyon

**Affiliations:** Department of Biology, University of West Georgia, Carrollton, GA 30118, USA; E-Mails: jrpittman@ega.edu (J.R.P.); johnsonlfall10@gmail.com (L.C.K.)

**Keywords:** carbon-starvation, stress response, stress resistance, starvation survival, strain variation, opportunistic pathogen

## Abstract

The broad host-range pathogen *Serratia marcescens* survives in diverse host and non-host environments, often enduring conditions in which the concentration of essential nutrients is growth-limiting. In such environments, carbon and energy source starvation (carbon-starvation) is one of the most common forms of stress encountered by *S. marcescens*. Related members of the family *Enterobacteriaceae* are known to undergo substantial changes in gene expression and physiology in response to the specific stress of carbon-starvation, enabling non-spore-forming cells to survive periods of prolonged starvation and exposure to other forms of stress (*i.e.*, starvation-induced cross-resistance). To determine if carbon-starvation also results in elevated levels of cross-resistance in *S. marcescens*, both log-phase and carbon-starved cultures, depleted of glucose before the onset of high cell-density stationary-phase, were grown in minimal media at either 30 °C or 37 °C and were then challenged for resistance to high temperature (50 °C), low pH (pH 2.8), and oxidative stress (15 mM H_2_O_2_). In general, carbon-starved cells exhibited a higher level of resistance to thermal stress, acid stress, and oxidative stress compared to log-phase cells. The extent of carbon-starvation-induced cross-resistance was dependent on incubation temperature and on the particular strain of *S. marcescens*. In addition, strain- and temperature-dependent variations in long-term starvation survival were also observed. The enhanced stress-resistance of starved *S. marcescens* cells could be an important factor in their survival and persistence in many non-host environments and within certain host microenvironments where the availability of carbon sources is suboptimal for growth.

## 1. Introduction

For many years, the Gram-negative bacterium *Serratia marcescens* was regarded as a generally harmless, saprophytic microorganism [1]. More recently, we have come to understand that *S. marcescens* is an emerging, broad host-range, opportunistic pathogen of growing importance [1,2,3,4]. Depending on the strain, *S. marcescens* can harbor genetic elements encoding virulence factors and antibiotic resistance determinants [5,6]. Reservoirs and sources of human infection frequently include nutrient-poor environments, such as saline solutions and contact lens solutions [7,8]. Starvation is the normal state of affairs for bacteria in such environments, and physiological mechanisms for coping with starvation stress are essential for bacterial survival under these conditions. In other closely related members of the family *Enterobacteriaceae*, such as *Escherichia coli* and *Salmonella enterica*, starvation stress is known to trigger a global change in gene expression and cellular physiology referred to as the starvation-stress response or SSR [9,10,11]. The response to carbon and energy starvation (carbon-starvation) is especially complex and involves induction of a network of genes ultimately leading to a distinctly different type of cell, the carbon-starved cell. In addition to being able to survive prolonged periods of carbon-starvation, starved cells develop cross-resistance toward a number of other environmental stresses including temperature stress, pH stress, and oxidative stress. Surprisingly little is known about the effects of starvation stress on *S. marcescens* despite potential connections to environmental survival and pathogenesis.

Environmental strains of *S. marcescens*, such as those isolated from soil and bodies of water, grow to form pink to red colored colonies on solid media due to the production of the pigment prodigiosin, while strains isolated from clinical specimens tend to be non-pigmented [12,13,14]. In addition, some strains produce less pigment when grown at temperatures above 25 °C–30 °C. When streaked onto rich agar media and incubated at room temperature, the type strain *Serratia marcescens* subsp. *marcescens* Bizio ATCC 13880, grows to form a mixture of pigmented, non-pigmented, and partially pigmented colonies. However, the differences between *S. marcescens* isolates are not limited to pigmentation. For example, temperature-dependent variations in metabolism, virulence, and antimicrobial resistance have also been observed [6,14,15,16,17,18,19,20].

*S. marcescens* is notorious for surviving on moist surfaces, in nutrient-poor aqueous environments, and in solutions containing powerful disinfectants [21,22,23,24,25,26,27]. In many of these situations, bacterial cells have to adapt to carbon-source availability. In a nosocomial setting, stress-resistant carbon-starved cells surviving on or in fomites are likely to be a source of infection, especially for immunosuppressed or immunocompromised patients. Once inside the human or animal host, phagocytic cells of the immune system may have difficulty in destroying stress-resistant carbon-starved cells [10,11,28]. Therefore, the physiological response to carbon-starvation is an important aspect of *S. marcescens* environmental survival and pathogenesis.

Elevated temperatures, acidic conditions, and exposure to oxidizing chemicals are also forms of stress *S. marcescens* confronts during passage through host and non-host environments, and any increase in the resistance of carbon-starved cells to these forms of stress (*i.e.*, starvation-induced cross-resistance) would influence the survival and spread of *S. marcescens* as well. It is conceivable that *S. marcescens* encounters these potentially lethal stresses during various cleaning practices, during certain medical procedures, during a high fever, during passage through stomach acid, or while in the hostile microenvironment of the phagolysosome.

In this study we performed a series of experiments designed to determine if *S. marcescens* cells starved of an exogenous source of carbon and energy (*i.e.*, glucose) in minimal media for a period of 24 h show increased levels of resistance to high temperature, low pH, and the oxidizing agent hydrogen peroxide (H_2_O_2_) in comparison to actively growing, log-phase cells. In the experiments described herein, cells were depleted of glucose at mid-log-phase and were subsequently diluted 10-fold for each stress challenge. Thus, the cell density was much lower than that in a typical stationary-phase culture and carbon-starvation was a single, defined stress. These conditions are in contrast to the multiple stresses (e.g., oxygen limitation, pH changes, *etc.*) simultaneously imposed on bacterial cells during high cell-density stationary-phase [10]. However, carbon-starvation is similar to stationary-phase with regard to the cessation of growth and the lowered metabolic state. Experiments were carried out at both 30 °C (intended to mimic a non-host environment or an invertebrate-host environment) and 37 °C (mammalian-host body temperature). In addition, we report the results of long-term carbon-starvation studies at both incubation temperatures. Comparisons are made between the type strain *S. marcescens* ATCC 13880 and other pigmented and non-pigmented *S. marcescens* strains in terms of carbon-starvation-induced cross-resistance and long-term carbon-starvation survival showing that the SSR of *S. marcescens* is both strain- and temperature-dependent.

## 2. Materials and Methods

### 2.1. Bacterial Strains, Growth Media, and Culture Conditions

The *Serratia marcescens* strains used in this study are shown in Table 1. The pigmented type strain *Serratia marcescens* subsp. *marcescens* Bizio (ATCC 13880) was obtained from the American Type Culture Collection and was the primary strain used in this study. The genome sequence of this strain has been recently published [29]. The non-pigmented, *Drosophila* isolate Db10 [30,31] was a gift from the lab of Dr. Jonathan J. Ewbank (Centre d’Immunologie de Marseille-Luminy, Université de la Méditerranée, Marseille, France). For isolation of the new pigmented strain UWG6, soil samples were plated on Violet Red Bile Glucose agar (VRBG agar, BD Difco™, Sparks, MD, USA), isolated colonies were then streaked onto DNase Test Agar with Toluidine Blue (Difco™), and those colonies which were positive for glucose fermentation and DNase activity, were identified to the species level using the Enterotube II system (BD BBL™, Becton, Sparks, MD, USA). Lysogeny agar (Difco™ LB agar, Miller) was used for the maintenance of stock cultures. M9 broth (Difco™ M9 minimal salts), with varying concentrations of glucose, was used for all experimental cultures. M9HiC media contained a standard concentration of glucose (0.4% w/v), M9LoC media contained a growth-limiting concentration of glucose (0.03% w/v), and M9NoC media had no glucose. All experimental cultures were incubated aerobically in a shaking incubator. Experiments were performed at either 30 °C or 37 °C from beginning to end unless otherwise noted (e.g., 50 °C for thermal challenges).

**Table 1:** Strains of *Serratia marcescens* used in this study.

Strain Designations	Source of Isolation	Pigmentation	Other Relevant Characteristics	References
ATCC 13880	Pond water	Mixed colony pigmentation at 30 °C; less pigmentation at 37 °C	Type strain of species; quality control strain; genome sequence complete	[3,29]
Db10	*Drosophila melanogaster*	No pigmentation at either 30 °C or 37 °C	Invertebrate pathogen used in studies with host *Caenorhabditis elegans*; genome sequence complete	[30,31]
UWG6	rhizosphere soil	Red pigmentation at 30 °C; pale pink at 37 °C	Positive for glucose fermentation and DNase activity; bile-resistant	This study

### 2.2. Starvation-Induced Cross-Resistance Challenges

Cross-resistance challenges were performed as described by Kenyon *et al.* [32] with minor changes regarding the growth medium, dilution buffer, and challenge parameters. Overnight cultures were prepared by inoculating 1 mL of M9HiC media with cells from a single, isolated colony on LB agar. Overnight cultures were incubated aerobically for 24 h at either 30 °C or 37 °C. The following day, 40 μL of overnight culture was transferred to 4 mL of either M9HiC or M9LoC media (1:100 v/v dilution) and incubated aerobically by shaking at either 30 °C or 37 °C. Growth was monitored by taking optical density readings (OD_600_) every 30 min. Log-phase (LP) cells were from M9HiC cultures which had reached an OD_600_ of 0.3–0.4 (mid-log-phase; ~10^8^–10^9^ CFU/mL). In M9LoC cultures, glucose is depleted and growth stops, after approximately 4–5 h of incubation, at an equivalent OD in the range of 0.3–0.4. Carbon-starved (CS) cells were from M9LoC cultures, which were kept in the incubator for 24 h after growth had ceased (24-h starved cells).

Challenge cultures were prepared by diluting 10 μL of LP or CS cells in 990 μL of M9NoC media (M9 buffer) either pre-warmed to 50 °C, previously lowered to a pH of 2.8 by the addition of HCl, or containing 15 mM H_2_O_2_. The challenge conditions chosen were a balance between the level of the stress and the exposure time which allowed for direct comparisons of the survival of LP and CS cells at different incubation temperatures. Thermal challenge cultures were incubated in a 50 °C water bath without shaking. Acid and H_2_O_2_ challenge cultures were incubated in a shaking incubator at either 30 °C or 37 °C. At the beginning of the challenge (time zero) and at later, pre-determined time points, 10-μL samples were removed and serially diluted in microtiter plates containing 90 μL of M9 buffer per well to generate a 10-fold dilution series. Ten-microliters from each dilution was spotted onto LB agar in triplicate and plates were incubated at either 30 °C or 37 °C for 24 h. Colonies from suitable dilutions were counted and the number of CFU/mL in each challenge culture was determined. The percent survival relative to the initial CFU/mL for each challenge culture at time zero is shown.

### 2.3. Long-Term Carbon-Starvation Survival

Long-term starvation experiments were performed as described by Kenyon *et al.* [32] with minor changes regarding the growth medium and dilution buffer. Long-term carbon-starvation cultures were prepared by first growing cells to mid-log-phase in M9HiC media (LP cells) and then diluting them 10-fold in M9NoC media at either 30 °C or 37 °C (e.g., 2.5 mL of LP culture was transferred to 22.5 mL of M9NoC media in a 125-mL baffled culture flask). The carryover of residual glucose allows for the continued growth of the culture for an additional one or two days before glucose is exhausted [32]. Once glucose is depleted, growth of the culture ceases and cells begin to starve. Starvation cultures were incubated for a total of 21 days at either 30 °C or 37 °C. Samples were then removed and viability counts were determined as described above for cross-resistance challenges. Survival is shown as a percentage of the highest CFU/mL reached for each starvation culture (~10^8^–10^9^ CFU/mL).

### 2.4. Statistical Analysis of Survival Data

Percent survival data is reported as the mean ± the standard error from the mean (SEM) from at least three independent experiments. A Student’s *t*-test was performed to determine if differences between percent survival values were statistically significant. A *p*-value of < 0.05 was considered significant.

## 3. Results

### 3.1. Starvation-Induced Cross-Resistance to Thermal Stress in S. marcescens ATCC 13880

**Figure 1:**
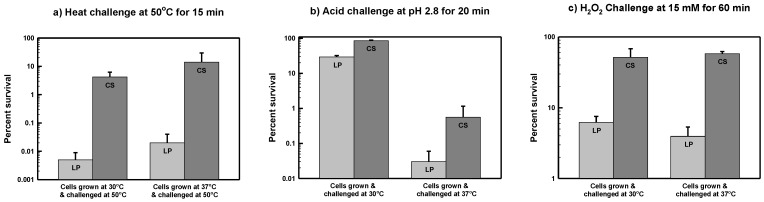
Starvation-induced cross-resistance in *Serratia marcescens* ATCC 13880. For thermal stress challenges (**a**), log-phase (LP) and carbon-starved (CS) cells of *S. marcescens* ATCC 13880 were grown at either 30 °C or 37 °C and then challenged at 50 °C for 15 min as described in Materials and Methods. For the acid stress and oxidative stress challenges (**b**,**c**), LP and CS cells of *S. marcescens* ATCC 13880 at either 30 °C or 37 °C and then challenged (pH 2.8 for 20 min or 15 mM H_2_O_2_ for 60 min) at the same temperature (**b**,**c**). Survival is reported as a percentage of the initial viability of the culture (*i.e.*, CFU/mL) at the beginning of the challenge experiment (*i.e.*, at time zero). Results are shown as the Mean ± SEM calculated from at least three independent experiments.

A marked difference between the thermotolerance of log-phase and carbon-starved *Serratia marcescens* ATCC 13880 cultures was observed (Figure 1a). After 15 min at 50 °C, the percent survival of LP cells grown at 30 °C dropped several order of magnitude relative to the maximum viability at time zero (0.005% of maximum). In comparison, the survival of 24-h CS cells declined to 4.2% of maximum. There was a difference in survival of more than 800-fold between LP and CS cells.

Cells grown at 37 °C showed roughly equivalent levels of survival, and there was a similar difference between the percent survival of LP and CS cells grown at this temperature and then challenged in the same manner (Figure 1a). A general trend toward greater thermotolerance was noted for both LP and CS cells grown at 37 °C, although the data presented in Figure 1 do not support statistically significant differences between cultures grown at 30 °C *versus* 37 °C (*p* > 0.05). In summary, non-growing, starved *S. marcescens* cells are far more resistant to thermal stress in comparison to actively growing cells.

### 3.2. Starvation-Induced Cross-Resistance to Acid Stress in S. marcescens ATCC 13880

Both LP and CS *S. marcescens* ATCC 13880 cells grown at 30 °C and then exposed to acid pH at the same temperature showed a high level of resistance (Figure 1b). After a 20-min exposure to pH 2.8, the viability of LP cells only fell to about 30% of the maximum level at time zero. Although LP and CS cells grown at 30 °C were roughly comparable in their level of acid resistance, CS cells still fared better with approximately 85% of cells remaining viable after 20 min of acid challenge (*p* < 0.05).

The overall level of acid resistance was much less for *S. marcescens* cells grown and challenged at 37 °C as shown in Figure 1b. Although the percent survival of CS cells was greater than that of LP cells (0.6% *vs.* 0.03%, respectively) the difference was not statistically significant (*p* > 0.05). These results suggest that acidic conditions are more detrimental for the survival of *S. marcescens* at elevated temperatures.

### 3.3. Starvation-Induced Cross-Resistance to Oxidative Stress in S. marcescens ATCC 13880

For LP *S. marcescens* ATCC 13880 cells grown at 30 °C and then exposed to 15 mM H_2_O_2_ at the same temperature, 6.2% of cells were still alive after 60 min (Figure 1c). This is a relatively high level of survival, and perhaps, reflects a certain degree of innate resistance toward oxidative stress. For comparison, only 0.16% of LP *S. enterica* serovar Typhimurium cells survived an identical challenge (data not shown). Although LP cells were already somewhat resistant to H_2_O_2_, CS cells showed an elevated level of survival eight times higher (51% survival) than that of LP cells (6.2% survival) at 30 °C (*p* < 0.05). The H_2_O_2_ resistance of LP and CS cultures grown and challenged at 37 °C was not significantly different from that at 30 °C (Figure 1c).

### 3.4. Starvation-Induced Cross-Resistance in Other Pigmented and Non-Pigmented Strains

Table 2 shows the results of carbon-starvation-induced cross-resistance challenges with the pigmented *S. marcescens* strain UWG6 and the non-pigmented strain Db10. Although, the results were generally similar to those described for type strain ATCC 13880, both strain- and temperature-dependent variations in starvation-induced cross-resistance were observed. LP cells of strain UWG6 grown at 37 °C displayed a high level of thermotolerance relative to the other strains. Like strain ATCC 13880, strains UWG6 and Db10 were also more acid-resistant when grown and challenged at 30 °C versus 37 °C. And, LP cells of UWG6 exhibited a level of acid resistance greater than Db10 at 30 °C. In summary, all *S. marcescens* strains tested exhibited some degree of carbon-starvation-inducible resistance to heat, acid, and H_2_O_2_.

**Table 2:** Starvation-induced cross-resistance to thermal, acid, and oxidative stress in pigmented and non-pigmented *Serratia marcescens* strains ^a^.

**Thermal Challenge (15 min at 50 °C)**			
Strain UWG6		Strain Db10	
30 °C	37 °C	30 °C	37 °C
LP = 0.040 ± 0.026	LP = 7.5 ± 4.3	LP = 0.020 ± 0.020	LP = 0.21 ± 0.11
CS = 38 ± 10	CS = 35 ± 15	CS = 3.1 ± 3.4	CS = 44 ± 7.8
**Acid Challenge (20 min at pH 2.8)**			
Strain UWG6		Strain Db10	
30 °C	37 °C	30 °C	37 °C
LP = 50 ± 30	LP = 0.36 ± 0.33	LP = 2.3 ± 2.2	LP = 0.01 ± 0.01
CS = 72 ± 16	CS = 0.89 ± 0.087	CS = 42 ± 7.6	CS = 3.9 ± 2.2
**Hydrogen Peroxide Challenge (60 min at 15 mM)**			
Strain UWG6		Strain Db10	
30 °C	37 °C	30 °C	37 °C
LP = 2.0 ± 1.5	LP = 0.60 ± 0.28	LP = 7.9 ± 2.5	LP = 6.5 ± 5.0
CS = 7.9 ± 1.7	CS = 56 ± 22	CS = 45 ± 20	CS = 56 ± 13

^a^ Cross-resistance challenges were performed as described in Materials and Methods, and results are reported as percent survival ± SEM from at least three independent experiments.

### 3.5. Long-Term Carbon-Starvation Survival in Pigmented and Non-Pigmented Strains

**Figure 2:**
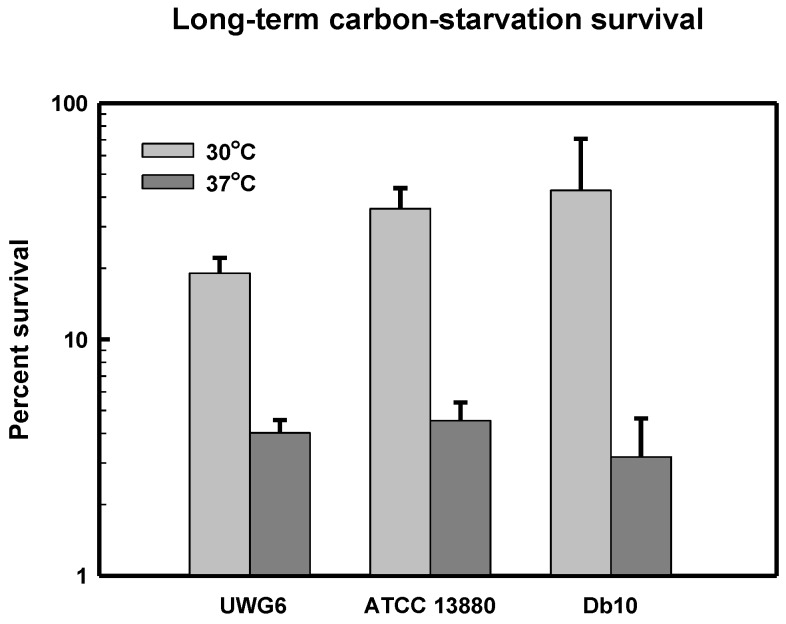
Long-term carbon-starvation survival of *Serratia marcescens* strains UWG6, ATCC 13880, and Db10. Cultures were grown and then starved of glucose in minimal media for a total of 21 days at either 30 °C or 37 °C as described in Materials and Methods. Survival is shown as a percentage of the highest viability (*i.e.*, CFU/mL) reached for that culture. Results are shown as the Mean ± SEM calculated from at least three independent experiments.

Cultures of *S. marcescens* strains UWG6, ATCC 13880, and Db10 were grown at either 30 °C or 37 °C and starved of carbon and energy (*i.e.*, glucose) at the same temperature over a three week time period. The results of the long-term carbon-starvation experiments are shown in Figure 2. The three *S. marcescens* strains exhibited comparable levels of long-term carbon-starvation survival at the two incubation temperatures, but all strains survived significantly better at 30 °C compared to 37 °C.

### 3.6. Stress-Induced Colony Phenotypes

Colonies of *S. marcescens* strains ATCC 13880 and UWG6 displayed unusual pigmentation phenotypes on LB agar following challenges for carbon-starvation-induced cross-resistance to thermal and acid stress and during long-term carbon-starvation experiments. These stress-induced colony (SIC) phenotypes are described in Table 3.

**Table 3:** Stress-induced colony (SIC) phenotypes of pigmented *Serratia marcescens* strains.

Strains	Type of Challenge	SIC Phenotypes
ATCC 13880	Acid challenge	pigmentation only in the center of some colonies (“fish eye” phenotype)
	Long-term carbon-starvation	non-pigmented, translucent colonies
UWG6	Thermal challenge	non-pigmented (white) colonies
	Acid challenge	non-pigmented (white to translucent) colonies
	Long-term carbon-starvation	translucent colonies; some with pink centers

## 4. Discussion

The goal of this study was to investigate whether non-growing, carbon-starved *S. marcescens* cells are more resistant to heat (50 °C), acid (pH 2.8), and H_2_O_2_ (15 mM) by performing cross-resistance challenges and directly comparing carbon-starved (CS) cells to growing, log-phase (LP) cells. To investigate the influence of growth temperature, experiments were carried out at either 30 °C or 37 °C. Cultures starved of glucose in minimal media for a period of 24 h, developed high levels of cross-resistance. The difference between LP and CS was greatest in terms of starvation-induced thermal resistance and was similar for the two pre-growth temperatures. Overall, CS cells were more resistant to acid stress than LP cells, but the level of acid resistance was temperature-dependent. At 30 °C, LP cells of the pigmented strains ATCC 13880 and UWG6 were highly resistant to acid pH, with survival values of 30% and 50%, respectively, compared to around 2% for type strain ATCC 13880. These survival values for *S. marcescens* can be compared to < 0.1% survival for LP cells of *S. enterica* serovar Typhimurium following an identical type of acid challenge experiment (data not shown). At 37 °C, both LP and CS cells of *S. marcescens* exhibited a much lower level of acid tolerance. The buffering capacity of prodigiosin is one possible explanation for this difference in acid tolerance, but it is also possible that other strain-specific differences play a role [33]. Furthermore, growth temperature is known to have an effect on prodigiosin biosynthesis but it also influences many other physiological processes (e.g., biosurfactant production) and has previously been shown to affect both stress resistance and virulence [20,34]. In terms of H_2_O_2_ resistance, CS cells survived much better than LP cells at both temperatures, although LP cells appeared to possess a certain level of inherent resistance, perhaps due to the constitutive expression of catalases and other protective factors specific to *S. marcescens*. Previous studies have reported that *S. marcescens* possesses its own collection of catalase enzymes and that H_2_O_2_ sensitivity is strain-dependent [35,36,37,38].

The long-term carbon-starvation survival of *S. marcescens* ATCC 13880 was appreciably higher at 30 °C compared to 37 °C (Figure 2), perhaps suggesting that the cellular damage caused by carbon-starvation is more severe at the elevated temperature. Interestingly, in thermal and acid cross-resistance challenges and in long-term carbon-starvation experiments, colonies, which grew on LB agar following stress exposure, frequently had aberrant morphologies. These stress-induced colonies (SICs) displayed phenotypic differences in both pigmentation and colony size. It has been known for some time that prodigiosin biosynthesis is sensitive to both low pH and high temperatures [39,40]. When subcultured on fresh LB agar, SICs typically reverted to the original colony morphologies. Starving cultures of pigmented strains ATCC 13880 and UWG6 in M9NoC, and colonies recovering on LB agar following starvation experiments, progressively lost pigmentation throughout the course of the long-term carbon-starvation experiment, possibly because prodigiosin was being catabolized as an intracellular reserve of carbon and energy. There have been many reports of stress-induced changes in *S. marcescens* colony morphologies over the years with strain-dependent variations in pigmentation and other phenotypic characteristics being a topic of interest for well over a century [1,2,3,4,41,42,43,44].

## 5. Conclusions

In conclusion, carbon-starved *S. marcescens* cells exhibit elevated levels of resistance to thermal, acid, and oxidative stress and survive long-term carbon-starvation for extended periods of time. These starvation-induced phenotypes are likely the culmination of numerous genetic and physiological changes occurring in direct response to both depletion of the carbon/energy source and growth arrest. This type of phenomenon is known as the starvation-stress response (SSR), and the SSR of *S. marcescens* could help to explain how this opportunistic pathogen is capable of persisting under nutrient-limiting environmental conditions while maintaining its virulence potential. Future research will address genetic mechanisms controlling the *S. marcescens* SSR. The general stress response sigma factor RpoS (σ^S^ or σ^38^) and the extracytoplasmic function sigma factor RpoE (σ^E^ or σ^24^) are regarded as master regulators of the SSR in several Gram-negative species [11,32,45,46,47,48], and polymorphisms in the *rpoS* chromosomal region are known to exist in enterobacteria and among strains of the same species, which could partially explain strain variations in the SSR [49,50]. Additional stress response networks controlled by regulatory complexes such as cAMP-CRP and ppGpp/DksA are likely to also contribute to the *S. marcescens* SSR as they do in other enteric species. Therefore, the transcriptional regulation of the *S. marcescens* SSR will be of future interest.
